# Optimal Pandemic Influenza Vaccine Allocation Strategies for the Canadian Population

**DOI:** 10.1371/journal.pone.0010520

**Published:** 2010-05-06

**Authors:** Ashleigh R. Tuite, David N. Fisman, Jeffrey C. Kwong, Amy L. Greer

**Affiliations:** 1 Department of Epidemiology, Dalla Lana School of Public Health, University of Toronto, Toronto, Ontario, Canada; 2 Department of Health Policy, Management and Evaluation, University of Toronto, Toronto, Ontario, Canada; 3 The Institute for Clinical Evaluative Sciences, Toronto, Ontario, Canada; 4 Modelling and Projection Section, Surveillance and Risk Assessment Division, Public Health Agency of Canada, Toronto, Ontario, Canada; INSERM, France

## Abstract

**Background:**

The world is currently confronting the first influenza pandemic of the 21^st^ century. Influenza vaccination is an effective preventive measure, but the unique epidemiological features of swine-origin influenza A (H1N1) (pH1N1) introduce uncertainty as to the best strategy for prioritization of vaccine allocation. We sought to determine optimal prioritization of vaccine distribution among different age and risk groups within the Canadian population, to minimize influenza-attributable morbidity and mortality.

**Methodology/Principal Findings:**

We developed a deterministic, age-structured compartmental model of influenza transmission, with key parameter values estimated from data collected during the initial phase of the epidemic in Ontario, Canada. We examined the effect of different vaccination strategies on attack rates, hospitalizations, intensive care unit admissions, and mortality. In all scenarios, prioritization of high-risk individuals (those with underlying chronic conditions and pregnant women), regardless of age, markedly decreased the frequency of severe outcomes. When individuals with underlying medical conditions were not prioritized and an age group-based approach was used, preferential vaccination of age groups at increased risk of severe outcomes following infection generally resulted in decreased mortality compared to targeting vaccine to age groups with higher transmission, at a cost of higher population-level attack rates. All simulations were sensitive to the timing of the epidemic peak in relation to vaccine availability, with vaccination having the greatest impact when it was implemented well in advance of the epidemic peak.

**Conclusions/Significance:**

Our model simulations suggest that vaccine should be allocated to high-risk groups, regardless of age, followed by age groups at increased risk of severe outcomes. Vaccination may significantly reduce influenza-attributable morbidity and mortality, but the benefits are dependent on epidemic dynamics, time for program roll-out, and vaccine uptake.

## Introduction

The rapid global spread of a novel swine-origin influenza A (H1N1) (pH1N1) virus led the World Health Organization to declare an influenza pandemic on June 11, 2009 [1]. When there is a good match between circulating and vaccine strains, influenza immunization is the most effective preventive measure for reducing influenza-related morbidity and mortality [2]. Development of a vaccine against pH1N1 began in the early phases of the epidemic, leading to questions about prioritization of vaccine allocation within populations, given that not all vaccine would be distributed at once (due to production and logistical constraints).

Seasonal influenza immunization campaigns typically target the elderly and those of any age with one or more underlying medical conditions, under the assumption that it is best to protect those most likely to have complications from influenza. Recently, there has been debate over whether this is the best approach [3], [4]. The degree of protection conferred by the influenza vaccine appears to be lower in the elderly than in the general population [5] and it has been suggested that an immunization strategy based on reducing transmission would have a greater impact on reducing overall disease burden than the current practice of focusing vaccination efforts on at-risk groups [6]. In particular, the potential benefit of preferentially vaccinating school-aged children has been discussed, since this age group is disproportionately responsible for influenza transmission [7], [8], [9].

As with earlier pandemics, pH1N1 is characterized by age distributions that are distinct from those observed in seasonal influenza epidemics, with higher attack rates and increased proportionate mortality, in younger individuals [10], [11], [12]. This differential vulnerability to infection by age will have important implications for the choice of optimal vaccination strategies [13].

Given the uncertainty surrounding optimal vaccine allocation strategies and the unique epidemiological characteristics of pH1N1, we sought to determine optimal prioritization of vaccine distribution among different age groups in order to minimize influenza-attributable morbidity and mortality in the Canadian population. To address this question we developed an age-structured mathematical model to describe expected pH1N1 transmission during the 2009–2010 influenza season. We used this model to evaluate the optimal sequencing of vaccination allocation strategies. Each strategy was tested using different assumptions relating to pre-existing immunity, vaccination coverage, and the timing of the epidemic peak. The outcomes of interest were influenza-attributable morbidity and mortality under different vaccination strategies.

## Methods

### Model structure

**Figure 1:**
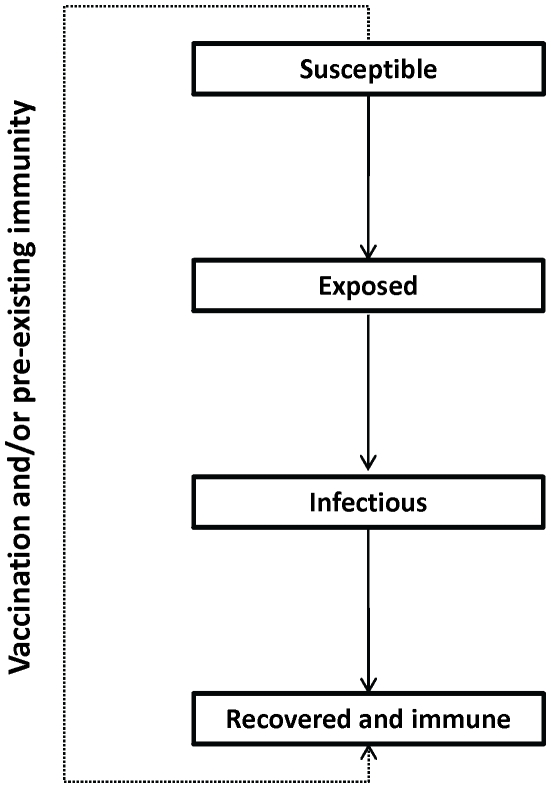
Outline of model structure, showing population flows between compartments. Each compartment is further stratified by age category (and by healthy and chronic condition states, where required).

We developed a deterministic, age-structured compartmental model of influenza transmission in the Canadian population (see **Figure 1** for overall structure and **File S1** for additional model details). The model ran from mid-April, 2009 (the date of the first identified cases of pH1N1 in Ontario, Canada) to June 30, 2010, representing a single influenza season. As a result, we did not consider waning immunity following infection or vaccination, migration into or out of the population, or population aging.

The population was divided into four compartments representing different disease states: susceptible (*S*), exposed (*E*; i.e., infected but not infectious), infectious (*I*), and recovered (*R*). Transmission of infection occurred through contact between susceptible and infectious individuals. We assumed that 40% of infections were asymptomatic [14], but did not consider differential transmission in symptomatic versus asymptomatic cases.

### Age structure and mixing patterns

To explore how vaccination of different age groups would impact overall influenza morbidity and mortality and to enable the representation of more realistic contact patterns within and between age groups, we included age stratification. The population was divided into seven age classes with the following cutoffs: 0–4, 5–13, 14–17, 18–22, 23–52, 53–64 and ≥65. Demographic information was obtained from 2006 Canadian census data [15]. We included the 53–64 year old age category to model the decreased susceptibility observed in persons born prior to the 1957 pandemic [12], [16], [17] and divided the younger ages according to school groupings to allow for the modeling of school-based vaccination programs. Mixing within and between age strata was based on a population-based prospective study of contact patterns in eight European countries [18].

For a subset of model scenarios, each age class was further subdivided into two states: healthy or underlying chronic medical condition for which seasonal influenza immunization is recommended. Transitions between model compartments were identical for individuals in the healthy or chronic condition states, but probabilities of experiencing severe clinical outcomes were different. We included a separate pregnancy state, representing women in the second or third trimester of pregnancy, with the number of women expected to be in this state at any given point in time derived using annual estimates of pregnancies and live births in Canada (see **File S1** for further details) [19], [20].

### Pre-existing immunity

To reflect the presence of immunity due to previous exposure to related influenza strains among individuals aged 53 and over, resistance to pH1N1 was modeled by moving some individuals from the susceptible to the resistant compartment at time zero. Since it is currently clinically impractical to distinguish individuals with pre-existing exposure to the circulating strains, we assumed that they received the same vaccination coverage as the susceptible population (i.e. there was no way to preferentially immunize the truly susceptible population).

### Vaccination

Vaccination with two doses of H1N1 vaccine was modeled by removing a select number of individuals from the susceptible compartment immediately following administration of the second dose of vaccine. Vaccination began in mid-November (November 15^th^), with a delay of 21 days between administration of the first and second doses. The fraction of the vaccinated population that acquired immunity was based on vaccine effectiveness estimates of 70 percent; for a given age group, with a vaccine effectiveness (*VE*) and coverage (*C*), the proportion removed from the susceptible to the resistant compartment was VE*C. We assumed that this group was fully protected against infection, with the remaining fraction VE*(1-*C*) receiving no protection. Although this does not reflect the true situation, where most vaccinated individuals will experience some degree of protection, this approach has been used previously and has been demonstrated to provide a reasonable model of partial efficacy [21]. We did not consider the effect of partial protection following the first dose. We assumed that it took four weeks to administer the first dose of vaccine to all age groups and vaccine allocation within each targeted sub-group (described below) occurred simultaneously at the beginning of each week (see **File S1** for additional details on timing of vaccination).

### Disease natural history and model parameterization

Model parameters for pH1N1 were based on the initial case data from the province of Ontario (**Table 1**) [22]. A range of estimates of the proportion of the population aged ≥53 with pre-existing immunity to pH1N1 influenza was derived from reported serological data [23], the relative risk of infection by age observed in Ontario [12] and model calibration to the Ontario epidemic curve. We considered pre-existing immunity levels of 30, 50, and 70 percent in this segment of the population.

**Table 1:** Model Parameter Values.

Variable	Age group	Value (range)	Source
**Total population size**	all	31,612,905	2006 Census [15]
**Latent period (days)**	all	3.5	Model calibration
**Duration of infectiousness (days)**	all	2.5	Model calibration
**Effective reproductive number**	all	1.3 (1.15–1.31)	Model calibration
**Proportion of population with pre-existing immunity**	≥53	0.5 (0.3–0.7)	MMWR, 2009 [23], Fisman et al., 2009 [12], model calibration
**Vaccine effectiveness**	<65	0.7	Centers for Disease Control and Prevention, 2008 [48]
	≥65	0.7 (0.3–0.7)	
**Proportion of population with high-risk conditions** a	0–4	0.10	Moran et al., 2009 [49]; Canadian Community Health Survey, 2007 [50]
	5–13	0.10	
	14–17	0.12	
	18–22	0.11	
	23–52	0.13	
	53–64	0.27	
	≥65	0.43	

a High-risk conditions include one or more of: asthma, emphysema, chronic obstructive pulmonary disease, diabetes, heart disease, cancer, and stroke.

Age-specific hospitalization, ICU admission, and mortality rates were calculated using data from Ontario's Integrated Public Health Information System (iPHIS), which collected information on all laboratory-confirmed cases of pH1N1 in the province reported between April 13 and June 21, 2009 (**Table 2**) [22]. To account for expected under-ascertainment of less severe cases, we multiplied the denominator (total cases) by a factor of ten when calculating hospitalization and case-fatality rates [24].

**Table 2:** Estimated Rates of Hospitalization, ICU Admission, and Mortality by Age and Risk Group for pH1N1 in Ontario, April to June 2009.

Outcome	Age group	All	Persons with high-risk conditions^a^	Persons without high-risk conditions
**Hospitalization rate (per 1,000 symptomatic persons)** b	0–4	13.2	22.1	7.5
	5–17	2.5	5.1	0.9
	18–52	4.3	9.1	1.0
	≥53	12.9	21.0	8.2
**Intensive care unit admission rate (per 1,000 hospitalized patients)**	0–4	0	0	0
	5–17	50.0	64.5	0
	18–52	196.1	227.3	0
	≥53	300.0	333.3	250.0
**Case-fatality rate (per 1,000 symptomatic persons)** b	0–4	0	0	0
	5–17	0.06	0	0.1
	18–52	0.25	0.6	0
	≥53	3.9	5.3	3.1

a High-risk conditions include one or more of: asthma, emphysema, chronic obstructive pulmonary disease, diabetes, heart disease, cancer, and stroke.

b Denominators were inflated 10-fold to account for expected underrepresentation of less severe pH1N1 cases among laboratory-confirmed cases.

We used vaccination coverage data for the province of Ontario [25], [26], which operates a universal influenza immunization program that provides influenza vaccine free of charge to the entire population aged six months or older, as a base case for H1N1 vaccine uptake (**Table 3**). Telephone survey data on willingness to accept pH1N1 vaccine in the province of Ontario obtained using the province's Rapid Risk Factor Surveillance System (RRFSS) [27] was used as an upper bound of vaccine uptake in the Canadian population (Ruth Sanderson, Ontario Agency for Health Protection and Promotion, personal communication). Age-specific data on underlying chronic conditions were obtained from the 2007 cycle of the Canadian Community Health Survey [25].

**Table 3:** Influenza Vaccination Coverage Levels.

Variable	Age group	Base case^a^	Upper bound^b^
**Proportion vaccinated**	0–4	0.26	0.60
	5–13	0.30	0.60
	14–17	0.31	0.60
	18–22	0.29	0.62
	23–52	0.29	0.54
	53–64	0.47	0.65
	≥65	0.75	0.75

a Source: Moran et al., 2009 [49]; Kwong et al., 2008 [26].

b Source: RRFSS module (Ontario Ministry of Health and Long-Term Care/Ontario Agency of Health Protection and Promotion).

To model the impact of assumptions about the dynamics of pH1N1 transmission over the course of the summer, where typical influenza seasonality and changes in contact patterns may reduce the basic reproductive number (R0), we modified R0 to generate differential timing of the peak of the epidemic curve. We considered the effect of different vaccination strategies when the epidemic peak occurred in October (no change in R0 over the summer), November (R0 decreases but remains above endemic levels from July to September), December (R0 = 1 from July to September), or January (R0 = 1 from July to October). We also adjusted R0 to account for different levels of pre-existing immunity to pH1N1 in the population (i.e., to give the same *effective* reproductive number under different immunity assumptions).

### Vaccination scenarios

We considered four vaccination strategies. For all scenarios, the total number of vaccine doses was not a limiting factor; adequate supply of vaccine was available for all individuals requiring immunization [28].

- Age-attack rate-based strategy (AR): Vaccine distributed first to age groups with the highest model-predicted attack rates (order of vaccine allocation by age group: 5–17, 18–52, 0–4, ≥53)
- Age-outcome-based strategy (Outcome): Vaccine distributed first to age groups at the highest risk of a severe outcome, defined as hospitalization, ICU admission, or death, following infection with pH1N1, ranked in order of probability of death, ICU admission, and hospitalization (order of vaccine allocation by age group: ≥53, 18–52, 0–4, 5–17).
- and (iv) Risk-based strategy (High risk/AR or High risk/Outcome): Vaccine preferentially distributed to individuals of any age with an underlying risk condition (based on seasonal influenza recommendations [2]) and pregnant women (in the second or third trimester), followed by an attack rate- or outcome-based strategy described above (i.e., based on age-group ranking) (delayed by one week to allow for immunization of high-risk individuals first).

### Model calibration

The model was calibrated to fit the initial epidemic curve observed in Ontario (by minimizing the sum-of-squares difference between model projections and the observed epidemic curve). Data for laboratory-confirmed cases with a reported exposure date between April 13 and June 1, 2009 were obtained from iPHIS. Travel history data, including illness on return to Mexico, were used to model the observed multiple introductions of pH1N1 into the Ontario population early on in the pandemic.

### Sensitivity analyses

We tested the robustness of model projections to baseline assumptions and parameter values by performing sensitivity analyses, with model inputs varied over plausible ranges, and incorporating alternate assumptions regarding vaccine program attributes. We evaluated the effect on model outputs of changing the time period for delivery of the first dose of vaccine to the entire population to two or six weeks, switching to a single vaccine dose, reducing vaccine effectiveness in the ≥65 age group (across a range of effectiveness of 30–60 percent), and varying the proportion of asymptomatic cases. We also assessed the impact of using alternate estimates of hospitalizations, ICU admissions, and mortality derived from the U.S. population [29], limiting prioritization of individuals with underlying medical conditions in the risk-based strategies to those under 65 years of age, and applying a limited vaccine supply scenario.

## Results

### Initial epidemic dynamics and model calibration

**Figure 2:**
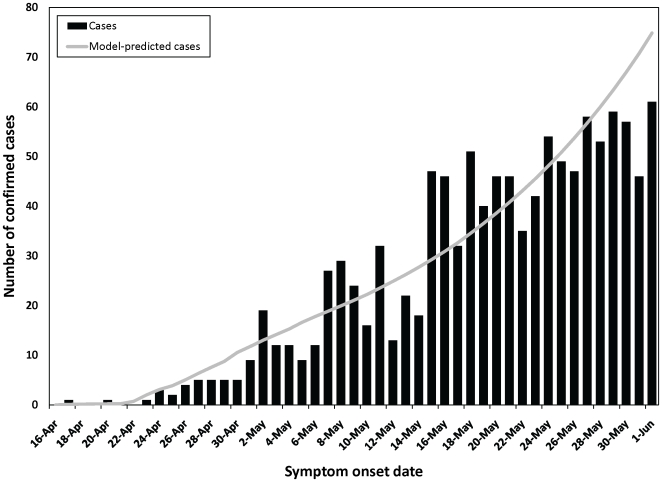
Confirmed cases of locally-acquired pH1N1 in Ontario by symptom onset date, April 16–June 1, 2009. Cases that reported a history of travel to Mexico prior to illness onset are not included. Model-predicted cases assuming 50 percent pre-existing immunity in the ≥53 age group, Re of 1.3, latent period of 3.5 days, and duration of infectiousness of 2.5 days are shown (line).

**Figure 3:**
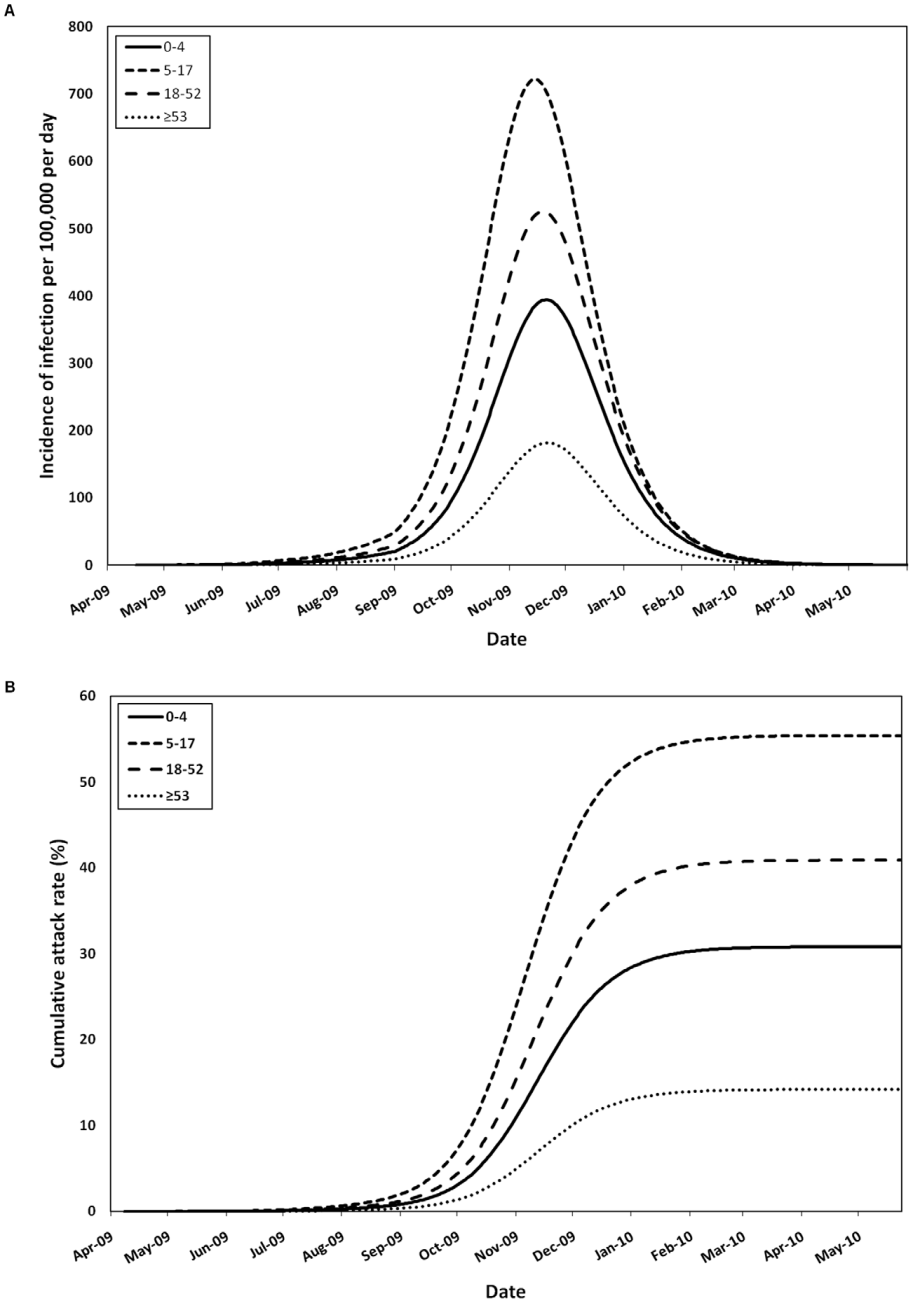
Model-predicted pH1N1 infection dynamics in the absence of vaccination. (**A**) Simulated age-stratified daily pH1N1 infection incidence per 100,000 population and (**B**) age-specific attack rates between April 2009 and June 2010, in the absence of vaccination or other interventions. Both symptomatic and asymptomatic cases are shown. The curves are based on an assumption of fifty percent pre-existing immunity in the ≥53 age group and a decrease in Re from 1.3 to 1.15 between July and September.

The model appeared well-calibrated to epidemic curves for pH1N1 influenza and matched the initial transmission dynamics observed in Ontario (**Figure 2**). **Figure 3** illustrates the pH1N1 infection dynamics generated by the model; epidemic curves peaked in different months, depending on assumptions made about influenza transmission behaviour during the summer months, but overall attack rates were consistent across model runs for a given estimate of pre-existing immunity in the pre-1957 cohort. In the absence of vaccination, the average infection attack rate across the entire Canadian population was 35.1% (range 33.2–36.8%). Age-specific patterns of influenza transmission reflected typical mixing patterns within a population, with epidemic curves peaking first in younger age groups, followed by the elderly.

### Effect of timing of epidemic peak in relation to vaccine availability on outcomes

**Figure 4:**
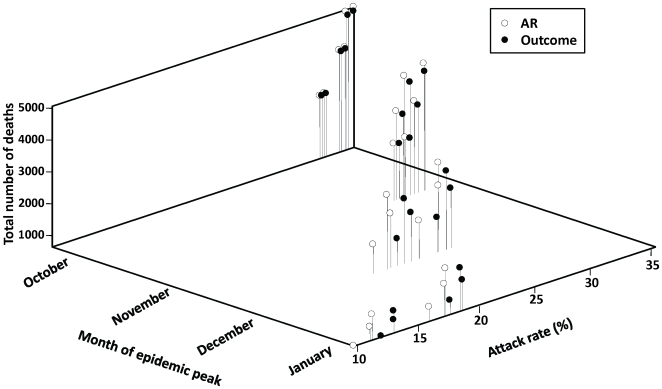
Effect of timing of epidemic peak on preferred vaccination strategy. Total model-predicted attack rates and deaths by month of the pandemic peak are shown, when implementing attack rate (AR)- or outcome-based vaccination strategies. For each month of the epidemic peak, outcomes are presented for three values of pre-existing immunity among individuals aged ≥53 (30%, 50%, and 70%) and two vaccination coverage levels (base case and upper bound). For all scenarios, vaccination campaigns are initiated on November 15, 2009.

Given the uncertainty around pH1N1 dynamics and timelines for vaccine delivery, we investigated the impact of the timing of the epidemic peak on whether an attack rate- or outcome-based vaccination strategy was preferred (**Figure 4**). For an October peak, neither approach was likely to significantly alter outcomes. For each month that the epidemic was delayed, there was enhanced effectiveness of all vaccination strategies.

### Attack rate-based versus outcome-based vaccination strategies

**Figure 5:**
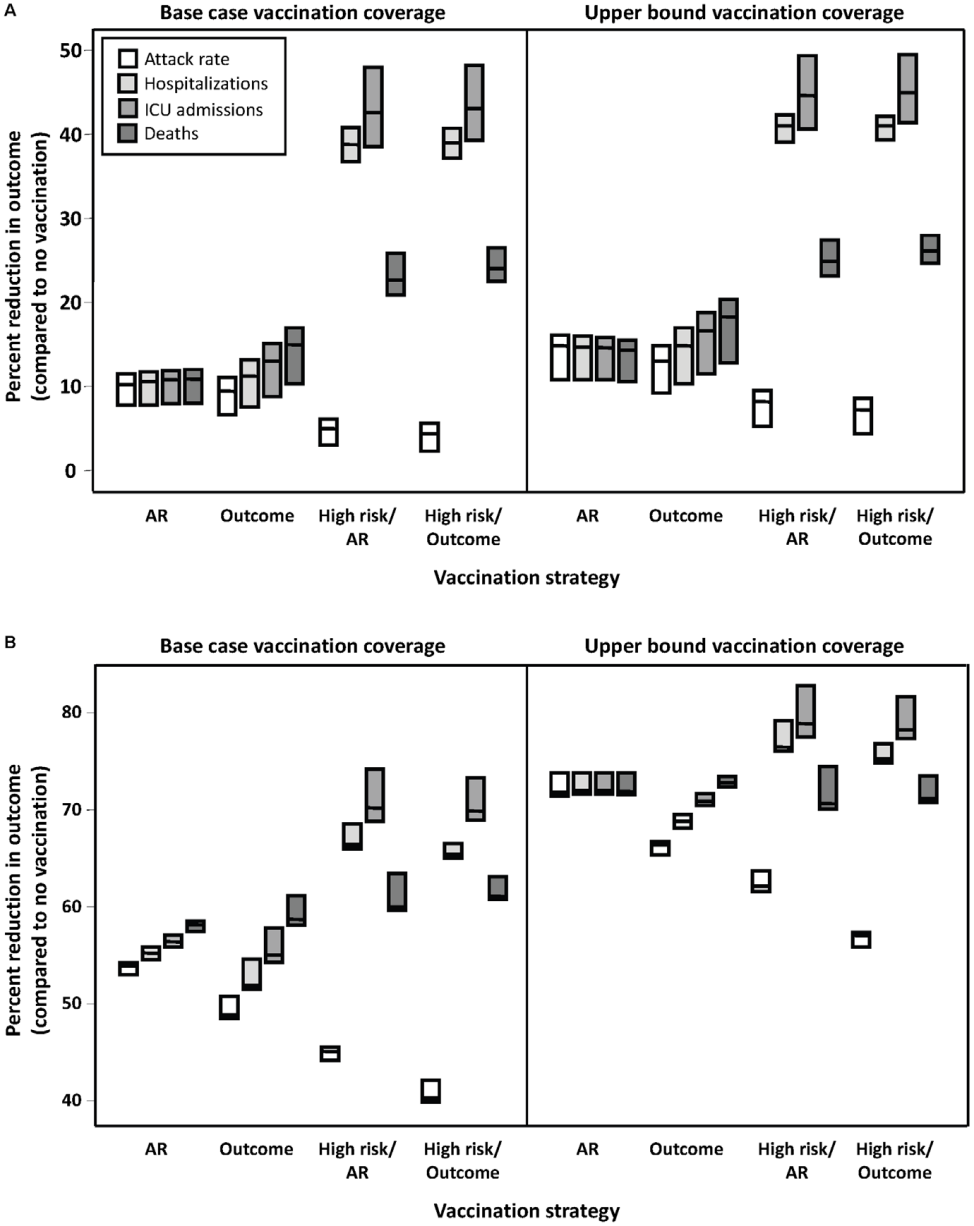
Impact of vaccination strategy on model outcomes. Percent reduction in attack rate, hospitalizations, ICU admissions, and total deaths, relative to no vaccination, under different vaccination strategies. The effectiveness of different strategies was evaluated assuming an epidemic peak in (**A**) November, 2009 or (**B**) January, 2010, with vaccination campaigns initiated on November 15, 2009. Results for October, 2009 and December, 2009 were similar to November, 2009 and January, 2010, respectively, and are not shown. The impact of vaccination coverage is also shown, with base case rates representing the lower bound of vaccine uptake in the Canadian population, compared to likely upper limits of vaccine uptake. The midpoint of the boxes represents the median percent reduction in the outcome of interest, with the upper and lower bounds representing the maximum and minimum reductions, respectively, under varying assumptions of pre-existing immunity in individuals aged ≥53 (i.e., 30%, 50%, or 70%). Details of the different vaccination strategies (AR, Outcome, High risk/AR, High risk/Outcome) are outlined in the Methods.

We evaluated the percent reduction in predicted attack rates, number of hospitalizations, ICU admissions, and deaths under these two strategies, relative to the no intervention scenario (**Figure 5**). The attack rate-based strategy was most effective at reducing the total number of infections and minimizing hospitalizations when the epidemic peaked in December or January, with minimal difference in the impact of competing strategies on overall attack rates when the epidemic peaked earlier. When there was 30 percent pre-existing immunity in the individuals born prior to 1957 group, there was no preferred strategy for minimizing hospitalizations. Using ICU admissions as the outcome of interest, the outcome-based strategy was preferred when there were low levels of pre-existing immunity, but there was no advantage to using one strategy over the other when immunity in the older age groups was ≥50 percent. By contrast, when mortality was assessed as the endpoint of interest, an outcome-based strategy was preferred to an attack rate-based strategy for any combination of values for pre-existing immunity and vaccine coverage, with the exception of the assumption of 70 percent immunity to pH1N1 in individuals aged ≥53 combined with a January peak. Under this latter scenario, there was no difference between strategies.

### Prioritization of vaccine delivery to individuals with underlying high-risk conditions (risk-based strategy)

We assessed the effect of preferentially immunizing individuals of any age with an underlying medical condition (representing 19% of the Canadian population), prior to implementing an attack rate- or outcome-based strategy. Despite the resulting delay in vaccine allocation to the remaining population, for all scenarios, hospitalizations and ICU admissions were reduced compared to vaccination strategies that did not target high-risk groups (**Figure 5**). When the epidemic peak occurred in December or January, this approach had a less marked effect in reducing mortality and resulted in higher cumulative attack rates than strategies that did not prioritize high-risk groups, whereas for an October or November epidemic peak, this approach had a larger effect in reducing mortality.

### Sensitivity analyses

Increasing the length of time to administer the first vaccine dose in all age groups from two to six weeks decreased the effectiveness of vaccination programs when the epidemic peak was in December or January, but did not have an effect when the peak occurred earlier (**Figure S1**).

Reducing vaccine effectiveness in individuals aged ≥65 did not have a marked effect on the ranking of vaccination strategies when levels of pre-existing immunity in the pre-1957 group were 30 or 50 percent. With 70 percent pre-existing immunity and lower bound vaccination coverage, reducing vaccine effectiveness to 60 percent or lower resulted in the attack rate-based strategy becoming favoured over outcome-based, when evaluating total deaths as the outcome of interest. Lowering vaccine effectiveness did not alter the ranking of the strategies when other outcomes (ICU admissions, hospitalizations, or attack rate) were the endpoints of interest.

Increasing the proportion of infectious individuals with asymptomatic influenza reduced the absolute number of cases experiencing severe outcomes, but did not change the relative rankings of the different strategies.

Emerging data suggest that a single dose of vaccine may be sufficient to confer protective immunity against infection with pH1N1 [30], [31]. When we tested the impact of a single dose on outcomes, we found no qualitative differences in the rank-order of vaccination strategies under different conditions for the majority of scenarios. However, for a January epidemic peak with high vaccination coverage, the attack rate-based strategy was more attractive than the outcome-based strategy, regardless of which endpoint was evaluated (**Figure S2**).

We considered a modified risk-based strategy, where only individuals aged less than 65 with underlying medical conditions were prioritized to receive vaccine (versus individuals of any age with underlying conditions in the main analysis) prior to implementing an attack rate- or outcome-based allocation strategy. For the majority of scenarios, there were no marked differences in outcomes, suggesting that excluding the ≥65 age group from vaccination prioritization schemes would not substantively alter the occurrence of poor outcomes. One notable exception arose when the epidemic peak occurred in November; in these scenarios, excluding individuals aged ≥65 age with chronic conditions resulted in greater mortality than when all individuals with chronic conditions were included.

When the total number of vaccine doses was limited to 20% of the population (enough vaccine to immunize approximately 6 million individuals, with doses divided equally over each week of vaccination campaign), there was no marked difference in results, compared to the other vaccination coverages considered, for an early epidemic peak (**Figure S3**). In contrast with our main analysis, an attack rate-based strategy was preferred over an outcome-based one for all outcomes evaluated when the epidemic peak occurred later (December or January), relative to when vaccine became available. For later epidemic peaks, there was no change in the order of preferred strategies when the risk-based strategies were evaluated.

The estimated risks of hospitalization, ICU admission, and mortality were calculated early in the pandemic. To assess the sensitivity of our results to these values, we repeated our analysis using more recent estimates derived from the U.S. population [29]. We projected hospitalization, ICU admission, and mortality using both the upper and lower bound estimates presented by Presanis and colleagues [29] (see **Figure S4** for results using upper bound estimates, similar patterns were observed using lower bound estimates, results not shown). Using these rates, vaccine prioritization preferences are unchanged for an early epidemic peak. However, for a later epidemic peak, an attack rate-based strategy resulted in a greater reduction in all outcomes under consideration, relative to an outcome-based strategy. This contrasts with our main results, where the preference in strategy for minimizing ICU admissions and mortality was dependent on underlying model assumptions. Given the absence of data on the frequency of occurrence of different outcomes in persons with or without underlying medical conditions (as defined in our analysis), we did not include the risk-based strategies in this sensitivity analysis.

## Discussion

We used a mathematical model to evaluate optimal pH1N1 vaccination strategies, focusing our analysis on the Canadian population and considering the effect of targeting different age groups for prioritization of vaccine allocation on projected hospitalizations and mortality. Depending on the outcome assessed and the assumptions used, both attack rate- and outcome-based strategies were effective in reducing morbidity and mortality, but in most scenarios, delaying vaccine distribution by one week to preferentially immunize individuals with underlying high-risk conditions was the optimal strategy. We observed that the dynamics of pH1N1 transmission is a critical area of uncertainty, with all vaccination strategies having limited impact if the epidemic peak occurs prior to or concomitantly with vaccine availability (projected for mid-November in our model).

Our analysis focused on the occurrence of severe outcomes and did not directly consider the effect of vaccination on reducing disease transmission and the resultant downstream effects, such as reduced societal disruption and economic costs (such as those associated with time lost from work or school). Additionally, when assessing severe outcomes, there is a need to consider how these outcomes may interact; for instance, a strategy that focuses on reducing mortality at the expense of higher attack rates could lead to the saturation of ICU capacity, resulting in higher mortality in younger age groups than has been observed to date.

The epidemiology of pH1N1 appears distinct from that of seasonal influenza (but similar to that of prior pandemics [11], [32]) in that younger age is associated with the highest attack rates, a phenomenon that has resulted in a higher absolute burden of morbidity and mortality in this age group than is typically observed with seasonal influenza, even though per-case risks of poor outcome may not differ from those seen with seasonal influenza. However, although older age groups are less likely to be infected with the pandemic strain than younger individuals, infections in individuals aged >50 years documented in Ontario have been associated with increased ICU admissions and death [22].

Several mathematical models have been developed to evaluate optimal vaccination strategies for pH1N1 [33], [34], [35], [36]. Among the key findings of these models are: the importance of early vaccination [36] and the role that prioritizing age groups based on patterns of severe outcomes can have in mitigating influenza impact in the population [34], [37]. Based on our results, preferential immunization of children, which has been recommended for both seasonal [6] and pandemic [33] influenza, and is represented by the attack rate-based strategy in our model, is the preferred strategy only when vaccine is available well in advance of the epidemic peak and its effectiveness is dependent on underlying model assumptions. By contrast, preferential vaccination of individuals with underlying medical conditions, regardless of age, was consistently observed to be an effective strategy to minimize hospitalizations, ICU admissions, and deaths attributable to pH1N1. Emerging empirical data from trials using both live attenuated [38] and inactivated [39] influenza vaccines support the focus on vaccination of younger individuals as a means to prevent infection in older individuals. Given that pH1N1 is likely to be the dominant seasonal strain in coming influenza seasons and, unlike the pandemic situation, seasonal influenza vaccines are generally available prior to surges in influenza activity, this paradigm may have application to near-term seasonal influenza seasons as well.

Our model assumes that two doses of pH1N1 vaccine will be required to elicit a protective response, but emerging data have demonstrated that a single dose may be sufficiently immunogenic in adults and children [30], [31], [40]. The implications of a single dose vaccine are similar to shifting the epidemic peak to later in the winter, resulting in enhanced effectiveness for any vaccination strategy adopted, relative to a two-dose schedule. The preference for an attack rate-based strategy using a single vaccine dose when vaccination coverage is high agrees with a recent study suggesting that targeting age groups at the highest risk of infection may be the optimal solution[33], but in our model, this is only the case when vaccine is available well before the epidemic peak. Finally, we evaluated the impact of poor vaccine effectiveness in older individuals on preferred strategies, as this has been a concern with seasonal vaccine [41]; we found limited impact of decreased effectiveness on the rank-ordering of preferred strategies except when older individuals were highly likely (70%) to be immune to infection in the absence of vaccination, and were effectively “pre-vacccinated”by early life influenza exposures.

Our analysis is subject to several important limitations. As with all mathematic models, this model includes simplifying assumptions and incorporates parameter values that are subject to some uncertainty. Model calibration to existing data was used to derive estimates of key epidemiologic parameters and these values are in agreement with estimates from other settings [42], [43]. We incorporated non-homogeneous mixing patterns between age groups, but did not consider the effect of spatial heterogeneity. However, other studies have demonstrated that estimates of R0 appear to be consistent across locations and spatial scales [44], [45]. Some other simplifying assumptions included non-differential transmissibility of influenza by symptomatic and asymptomatic cases and non-incorporation of other concurrent mitigation strategies on influenza transmission, including antivirals and social distancing measures, on influenza transmission. We also did not consider the impact of co-circulating seasonal influenza strains, although recent data suggest that reduced circulation of seasonal strains may be observed during a pandemic situation [46], [47]. To address the uncertainty in our estimates of mortality and hospitalization rates, due to both the low frequency of occurrence of these outcomes and reporting biases and other limitations inherent in surveillance data, we have focused our analysis on qualitative results. We have also included alternate estimates of these outcomes (derived from the U.S. population).

In summary, we have developed an age-structured mathematical model to evaluate optimal vaccination strategies for pH1N1. This model demonstrates the importance of the interaction between pH1N1 transmission dynamics and the demographic characteristics of population at risk of pH1N1 infection on the potential effectiveness of vaccination strategies. It also highlights the value of moving away from strictly age-based vaccination prioritization schemes toward strategies that target high-risk groups, regardless of age.

## Supporting Information

File S1 Mathematical model details
(0.30 MB PDF)

Figure S1 Percent reduction in attack rate, hospitalizations, ICU admissions, and total deaths, relative to no vaccination, assuming different program roll-out lengths. Outcomes were assessed assuming that time to administration of a single dose of vaccine to all age groups was 2, 4, or 6 weeks, with vaccination campaigns commencing on November 15, 2009. Estimates are pooled across vaccination strategy used (attack rate- and outcome-based), vaccination coverage (base case and upper bound), and levels of pre-existing immunity in individuals aged ≥53 (30%, 50%, and 70%) and are shown by month of epidemic peak.
(0.50 MB TIF)

Figure S2 Impact of a single dose vaccination schedule on percent reduction in attack rate, hospitalizations, ICU admissions, and total deaths, relative to no vaccination. The effectiveness of different strategies was evaluated assuming an epidemic peak in (A) November, 2009 or (B) January, 2010. Results for October, 2009 and December, 2009 were similar to November, 2009 and January, 2010, respectively, and are not shown. Vaccination campaigns began on November 15, 2009, with vaccine conferring a protective effect immediately after administration of a single dose. The impact of vaccination coverage is also shown, with base case rates representing the lower bound of vaccine uptake in the Canadian population, compared to likely upper limits of vaccine uptake. The midpoint of the boxes represents the median percent reduction in the outcome of interest, with the upper and lower bounds representing the maximum and minimum reductions, respectively, under varying assumptions of pre-existing immunity in individuals aged ≥53 (i.e., 30%, 50%, or 70%). Details of the different vaccination strategies (AR, Outcome, High risk/AR, High risk/Outcome) are outlined in the Methods.
(0.67 MB TIF)

Figure S3 Impact of limited vaccine supply on percent reduction in attack rate, hospitalizations, ICU admissions, and death, relative to no vaccination. The effectiveness of different strategies was evaluated assuming an epidemic peak in November, 2009 or January, 2010, with vaccination campaigns initiated on November 15, 2009. Results for October, 2009 and December, 2009 were similar to November, 2009 and January, 2010, respectively, and are not shown. Enough vaccine was available to vaccinate six million individuals, with an equal number of doses available each week of the campaign. The midpoint of the boxes represents the median percent reduction in the outcome of interest, with the upper and lower bounds representing the maximum and minimum reductions, respectively, under varying assumptions of pre-existing immunity in individuals aged ≥53 (i.e., 30%, 50%, or 70%). Details of the different vaccination strategies (AR, Outcome, High risk/AR, High risk/Outcome) are outlined in the Methods.
(0.52 MB TIF)

Figure S4 Comparison of effectiveness of attack rate- and outcome-based strategies using alternate estimates of hospitalization, ICU admission, and mortality. Percent reduction in outcomes relative to no vaccination was evaluated assuming an epidemic peak in (A) November, 2009 or (B) January, 2010, with vaccination campaigns initiated on November 15, 2009. Resultsfor October, 2009 and December, 2009 were similar to November, 2009 and January, 2010, respectively, and are not shown. The impact of vaccination coverage is also shown, with base case rates representing the lower bound of vaccine uptake in the Canadian population, compared to likely upper limits of vaccine uptake. The midpoint of the boxes represents the median percent reduction in the outcome of interest, with the upper and lower bounds representing the maximum and minimum reductions, respectively, under varying assumptions of pre-existing immunity in individuals aged ≥53 (i.e., 30%, 50%, or 70%). Details of the vaccination strategies are outlined in the Methods.
(0.76 MB TIF)

## References

[1] Chan M (2009). World now at the start of 2009 influenza pandemic.. http://www.who.int/mediacentre/news/statements/2009/h1n1_pandemic_phase6_20090611/en/.

[2] National Advisory Committee on Immunization (2008). Statement on influenza vaccination for the 2008–2009 season. An Advisory Committee Statement (ACS).. Can Commun Dis Rep.

[3] Nichol KL, Nordin JD, Nelson DB, Mullooly JP, Hak E (2007). Effectiveness of influenza vaccine in the community-dwelling elderly.. N Engl J Med.

[4] Simonsen L, Taylor RJ, Viboud C, Miller MA, Jackson LA (2007). Mortality benefits of influenza vaccination in elderly people: an ongoing controversy.. Lancet Infect Dis.

[5] Goodwin K, Viboud C, Simonsen L (2006). Antibody response to influenza vaccination in the elderly: a quantitative review.. Vaccine.

[6] Galvani AP, Reluga TC, Chapman GB (2007). Long-standing influenza vaccination policy is in accord with individual self-interest but not with the utilitarian optimum.. Proc Natl Acad Sci U S A.

[7] Manzoli L, Schioppa F, Boccia A, Villari P (2007). The efficacy of influenza vaccine for healthy children: a meta-analysis evaluating potential sources of variation in efficacy estimates including study quality.. Pediatr Infect Dis J.

[8] Negri E, Colombo C, Giordano L, Groth N, Apolone G (2005). Influenza vaccine in healthy children: a meta-analysis.. Vaccine.

[9] Smith S, Demicheli V, Di Pietrantonj C, Harnden AR, Jefferson T (2006). Vaccines for preventing influenza in healthy children.. Cochrane Db Syst Rev (Online).

[10] Ahmed R, Oldstone MB, Palese P (2007). Protective immunity and susceptibility to infectious diseases: lessons from the 1918 influenza pandemic.. Nat Immunol.

[11] Miller MA, Viboud C, Balinska M, Simonsen L (2009). The signature features of influenza pandemics—implications for policy.. N Engl J Med.

[12] Fisman DN, Savage R, Gubbay J, Achonu C, Akwar H (2009). Older age and a reduced likelihood of 2009 H1N1 virus infection.. N Engl J Med.

[13] Miller MA, Viboud C, Olson DR, Grais RF, Rabaa MA (2008). Prioritization of influenza pandemic vaccination to minimize years of life lost.. J Infect Dis.

[14] Fraser C, Riley S, Anderson RM, Ferguson NM (2004). Factors that make an infectious disease outbreak controllable.. Proc Natl Acad Sci U S A.

[15] Statistics Canada (2007). Age and sex highlight tables, 2006 Census.. http://www12.statcan.gc.ca/census-recensement/2006/dp-pd/hlt/97-551/index.cfm?Lang=E.

[16] Chowell G, Bertozzi SM, Colchero MA, Lopez-Gatell H, Alpuche-Aranda C (2009). Severe respiratory disease concurrent with the circulation of H1N1 influenza.. N Engl J Med.

[17] Dawood FS, Jain S, Finelli L, Shaw MW, Lindstrom S (2009). Emergence of a novel swine-origin influenza A (H1N1) virus in humans.. N Engl J Med.

[18] Mossong J, Hens N, Jit M, Beutels P, Auranen K (2008). Social contacts and mixing patterns relevant to the spread of infectious diseases.. PLoS Med.

[19] Statistics Canada (2005). Pregnancy outcomes by age group (live births).. http://www40.statcan.gc.ca/l01/cst01/hlth65b-eng.htm.

[20] Statistics Canada (2005). Pregnancy outcomes by age group (total pregnancies).. http://www40.statcan.gc.ca/l01/cst01/hlth65a-eng.htm.

[21] Bansal S, Pourbohloul B, Meyers LA (2006). A comparative analysis of influenza vaccination programs.. PLoS Med.

[22] Tuite AR, Greer AL, Whelan M, Winter AL, Lee B (2010). Estimated epidemiologic parameters and morbidity associated with pandemic H1N1 influenza.. CMAJ.

[23] Centers for Disease Control and Prevention (2009). Serum cross-reactive antibody response to a novel influenza A (H1N1) virus after vaccination with seasonal influenza vaccine.. MMWR Morb Mortal Wkly Rep.

[24] Wilson N, Baker MG (2009). The emerging influenza pandemic: estimating the case fatality ratio.. Euro Surveill.

[25] Beland Y (2002). Canadian community health survey—methodological overview.. Health Rep.

[26] Kwong JC, Stukel TA, Lim J, McGeer AJ, Upshur RE (2008). The effect of universal influenza immunization on mortality and health care use.. PLoS Med.

[27] Bingle CL, Holowaty PH, Koren IE, Picard L, Stewart PJ (2005). An evaluation of the Ontario Rapid Risk Factor Surveillance System.. Can J Public Health.

[28] Public Health Agency of Canada (2009). Guidance on H1N1 flu vaccine sequencing.. http://www.phac-aspc.gc.ca/alert-alerte/h1n1/vacc/vacc-eng.php.

[29] Presanis AM, De Angelis D, Hagy A, Reed C, Riley S (2009). The severity of pandemic H1N1 influenza in the United States, from April to July 2009: a Bayesian analysis.. PLoS Med.

[30] Clark TW, Pareek M, Hoschler K, Dillon H, Nicholson KG (2009). Trial of 2009 influenza A (H1N1) monovalent MF59-adjuvanted vaccine.. N Engl J Med.

[31] Greenberg ME, Lai MH, Hartel GF, Wichems CH, Gittleson C (2009). Response to a monovalent 2009 influenza A (H1N1) vaccine.. N Engl J Med.

[32] Henderson DA, Courtney B, Inglesby TV, Toner E, Nuzzo JB (2009). Public health and medical responses to the 1957–58 influenza pandemic.. Biosecur Bioterror.

[33] Medlock J, Galvani AP (2009). Optimizing influenza vaccine distribution.. Science.

[34] Chowell G, Viboud C, Wang X, Bertozzi SM, Miller MA (2009). Adaptive vaccination strategies to mitigate pandemic influenza: Mexico as a case study.. PLoS One.

[35] Sypsa V, Pavlopoulou I, Hatzakis A (2009). Use of an inactivated vaccine in mitigating pandemic influenza A(H1N1) spread: a modelling study to assess the impact of vaccination timing and prioritisation strategies.. Euro Surveill.

[36] Gojovic MZ, Sander B, Fisman D, Krahn MD, Bauch CT (2009). Modelling mitigation strategies for pandemic (H1N1) 2009.. CMAJ.

[37] Medlock J, Meyers LA (2009). Optimizing allocation for a delayed influenza vaccination campaign.. PLoS Curr Influenza.

[38] King JC, Stoddard JJ, Gaglani MJ, Moore KA, Magder L (2006). Effectiveness of school-based influenza vaccination.. N Engl J Med.

[39] Loeb M, Russell ML, Moss L, Fonseca K, Fox J (2010). Effect of influenza vaccination of children on infection rates in Hutterite communities: a randomized trial.. JAMA.

[40] Nolan T, McVernon J, Skeljo M, Richmond P, Wadia U (2010). Immunogenicity of a monovalent 2009 influenza A(H1N1) vaccine in infants and children: a randomized trial.. JAMA.

[41] Simonsen L, Reichert TA, Viboud C, Blackwelder WC, Taylor RJ (2005). Impact of influenza vaccination on seasonal mortality in the US elderly population.. Arch Intern Med.

[42] Fraser C, Donnelly CA, Cauchemez S, Hanage WP, Van Kerkhove MD (2009). Pandemic potential of a strain of influenza A (H1N1): early findings.. Science.

[43] Pourbohloul B, Ahued A, Davoudi B, Meza R, Meyers LA (2009). Initial human transmission dynamics of the pandemic (H1N1) 2009 virus in North America.. Influenza Other Respi Viruses.

[44] Mills CE, Robins JM, Lipsitch M (2004). Transmissibility of 1918 pandemic influenza.. Nature.

[45] Viboud C, Bjornstad ON, Smith DL, Simonsen L, Miller MA (2006). Synchrony, waves, and spatial hierarchies in the spread of influenza.. Science.

[46] Uphoff H, Geis S, Gruber A, Hauri A (2009). What will the next influenza season bring about: seasonal influenza or the new A(H1N1)v? An analysis of German influenza surveillance data.. Euro Surveill.

[47] Kelly H, Grant K (2009). Interim analysis of pandemic influenza (H1N1) 2009 in Australia: surveillance trends, age of infection and effectiveness of seasonal vaccination.. Euro Surveill.

[48] Centers for Disease Control and Prevention (2008). Prevention and control of influenza: recommendations of the Advisory Committee on Immunization Practices (ACIP), 2008.. http://www.cdc.gov/mmwr/preview/mmwrhtml/rr57e717a1.htm.

[49] Moran K, Maaten S, Guttmann A, Northrup D, Kwong JC (2009). Influenza vaccination rates in Ontario children: implications for universal childhood vaccination policy.. Vaccine.

[50] Statistics Canada (2007). http://www.statcan.gc.ca/cgi-bin/imdb/p2SV.pl?Function=getSurvey&SurvId=3226&SurvVer=1&SDDS=3226&InstaId=15282&InstaVer=4&lang=en&db=imdb&adm=8&dis=2.

