# Supplementary material for: Optimal Pandemic Influenza Vaccine Allocation Strategies for the Canadian Population
Source: PLoS One. 2010 May 6;5(5):e10520. doi: 10.1371/journal.pone.0010520 (PMC2865540; doi:10.1371/journal.pone.0010520)
Supplement: File S1 — Mathematical model details (0.30 MB PDF) [file pone.0010520.s001.pdf]

Supplementary Material

Model details

$$\frac{dS_a}{dt} = -\lambda S_a$$

$$\frac{dE_a}{dt} = \lambda S_a - \varepsilon E_a$$

$$\frac{dI_a}{dt} = \varepsilon E_a - \gamma I_a$$

$$\frac{dR_a}{dt} = \gamma I_a$$

$S_a(t)$, $E_a(t)$, $I_a(t)$, and *$R_a(t)$* are the respective number of susceptible, exposed, infectious, and recovered individuals in age groups a = 1, 2, . . . , 7. The rate of progression to infectiousness is ε, the recovery rate is γ. Upon implementation of vaccination, the number of individuals moved from the susceptible to recovered class is calculated by: $V_E C_a S_a$, where V_E is vaccine efficacy and C_a is vaccine coverage.

The age groups are defined as follows:

1: 0 – 4
2: 5 – 13
3: 14 – 17
4: 18 – 22
5: 23 – 52
6: 53 – 64
7: ≥65

Force of infection is given by,

$$\lambda_a = \sum_{a=1}^{7} \frac{\phi_{a\alpha\prime} \beta I_{\alpha\prime}}{N}$$

where $\phi_{a\alpha\prime}$ is the contact rate for infective individuals of age group $\alpha'(I_{\alpha'})$ with susceptible individuals of age group a, β is the probability of transmission given contact (assumed to be independent of age and calculated based on the R_0 value in the absence of vaccination), and N is total population size.

When including high-risk conditions, the model is subdivided into a_h and a_c for each age category, representing the healthy and chronic condition groups. The number of individuals moved from the a_h to the a_c group at the beginning of the model run is given by: $P_{ac}S_a$, where P_{ac} is the proportion of the population with a high-risk condition for a given age group. For a given age category, contact rates are assumed to be identical for the healthy and chronic condition groups. The pregnancy state was initially populated by drawing 200,000 susceptible individuals (the number of women expected to be in their second or third trimester of pregnancy at any given point in time) from the 23-52 age group, regardless of chronic condition status. Individuals enter the pregnant state at a rate equal to the daily hazard of becoming pregnant (0.00008); they are drawn from the susceptible, exposed, infectious, and recovered compartments of the 23-52 age group and placed in the corresponding compartment of the pregnant state. Similarly, individuals leave the pregnant state at a rate equal to the daily hazard of giving birth (0.00055), are drawn from the susceptible, exposed, infectious, and recovered compartments, and are returned to the corresponding compartment for the 23-52 age group. Contact rates in the pregnant group are assumed to be the same as for individuals in the 23-52 age group and we used the outcome rates observed in the 18-52 age group with chronic conditions.

Pre-existing immunity to pH1N1 (age groups 6 and 7) is calculated at time zero:

$$S_a = S_a(1 - pR_a)$$

$$R_a = S_a pR_a$$

where pR_{ap} is probability of pre-existing immunity to pH1N1.

Vaccination is implemented beginning in mid-November for pH1N1 (depending on age group and vaccination strategy used):

$$S_a = S_a(1 - C_a VE_a)$$

$$R_a = R_a + S_a C_a VE_a$$

where VE_a is age-specific vaccine effectiveness and C_a is age-specific vaccination coverage for pH1N1 (described in **Table 3**).

All individuals targeted for vaccination under a given strategy are moved simultaneously to the recovered compartment. Under a two dose vaccination schedule, vaccinated individuals are moved to the recovered compartment immediately following administration of the second dose of vaccine (i.e., 21 days weeks after administration of the first dose):

Date of first dose/second dose	Groups targeted for vaccination			
	Attack rate	Outcome	High risk/Attack rate	High risk/Outcome
Nov. 15, 2009 / Dec. 6, 2009	2, 3	6, 7	Chronic: all ages	Chronic: all ages
Nov. 29, 2009 / Dec. 13, 2009	4, 5	4, 5	Healthy: 2, 3	Healthy: 6, 7
Dec. 6, 2009 / Dec. 20, 2009	1	1	Healthy: 4, 5	Healthy: 4, 5
Dec. 13, 2009 / Dec. 27, 2009	6, 7	2, 3	Healthy: 1	Healthy: 1
Dec. 20, 2009 / Jan. 3, 2010	_	_	Healthy: 6, 7	Healthy: 2, 3
