# Supplementary figures and images for: Optimal Pandemic Influenza Vaccine Allocation Strategies for the Canadian Population
Source: PLoS One. 2010 May 6;5(5):e10520. doi: 10.1371/journal.pone.0010520 (PMC2865540; doi:10.1371/journal.pone.0010520)

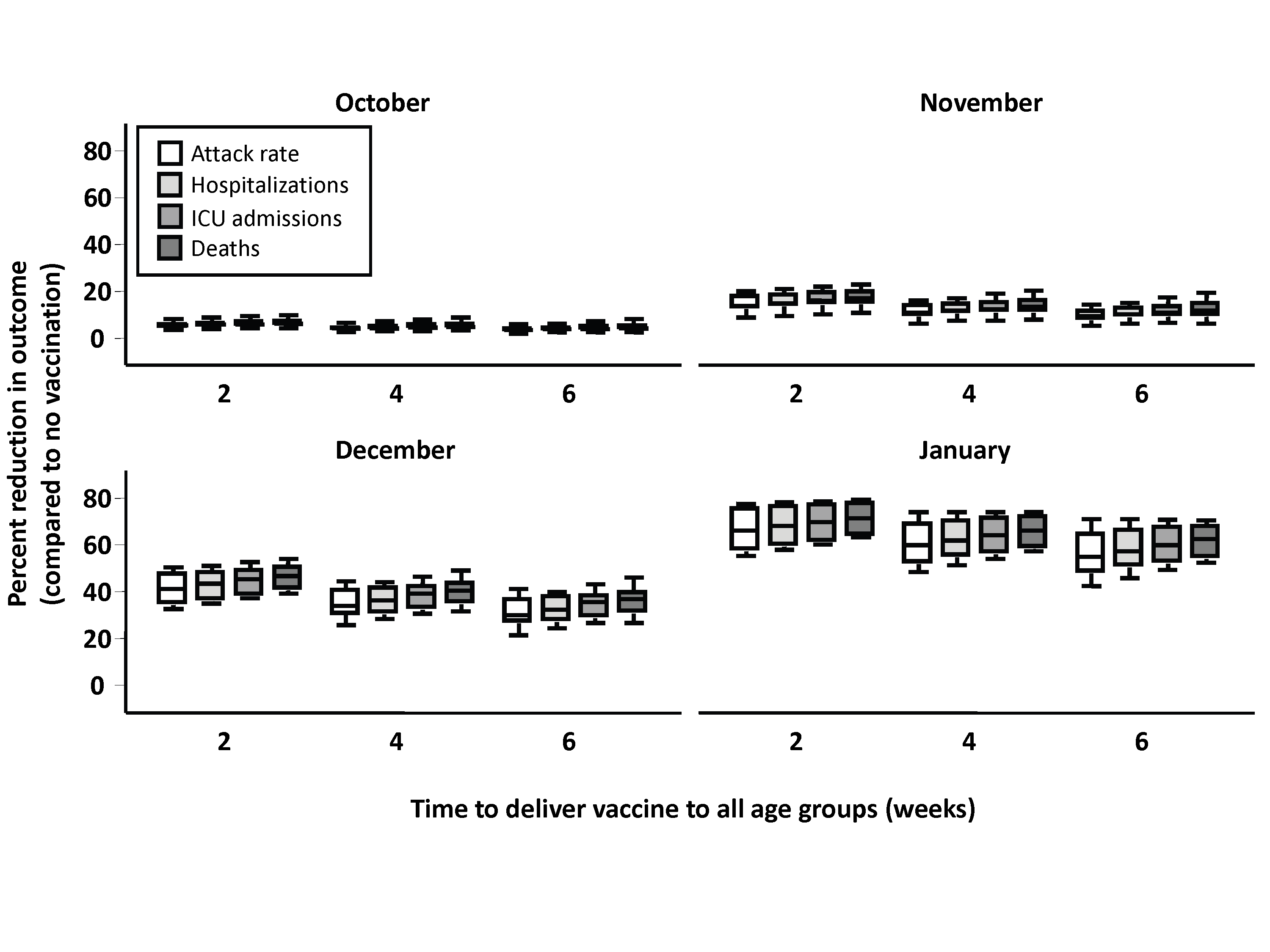

Supplement: Figure S1 — Percent reduction in attack rate, hospitalizations, ICU admissions, and total deaths, relative to no vaccination, assuming different program roll-out lengths. Outcomes were assessed assuming that time to administration of a single dose of vaccine to all age groups was 2, 4, or 6 weeks, with vaccination campaigns commencing on November 15, 2009. Estimates are pooled across vaccination strategy used (attack rate- and outcome-based), vaccination coverage (base case and upper bound), and levels of pre-existing immunity in individuals aged ≥53 (30%, 50%, and 70%) and are shown by month of epidemic peak. (0.50 MB TIF) [file pone.0010520.s002.tif]

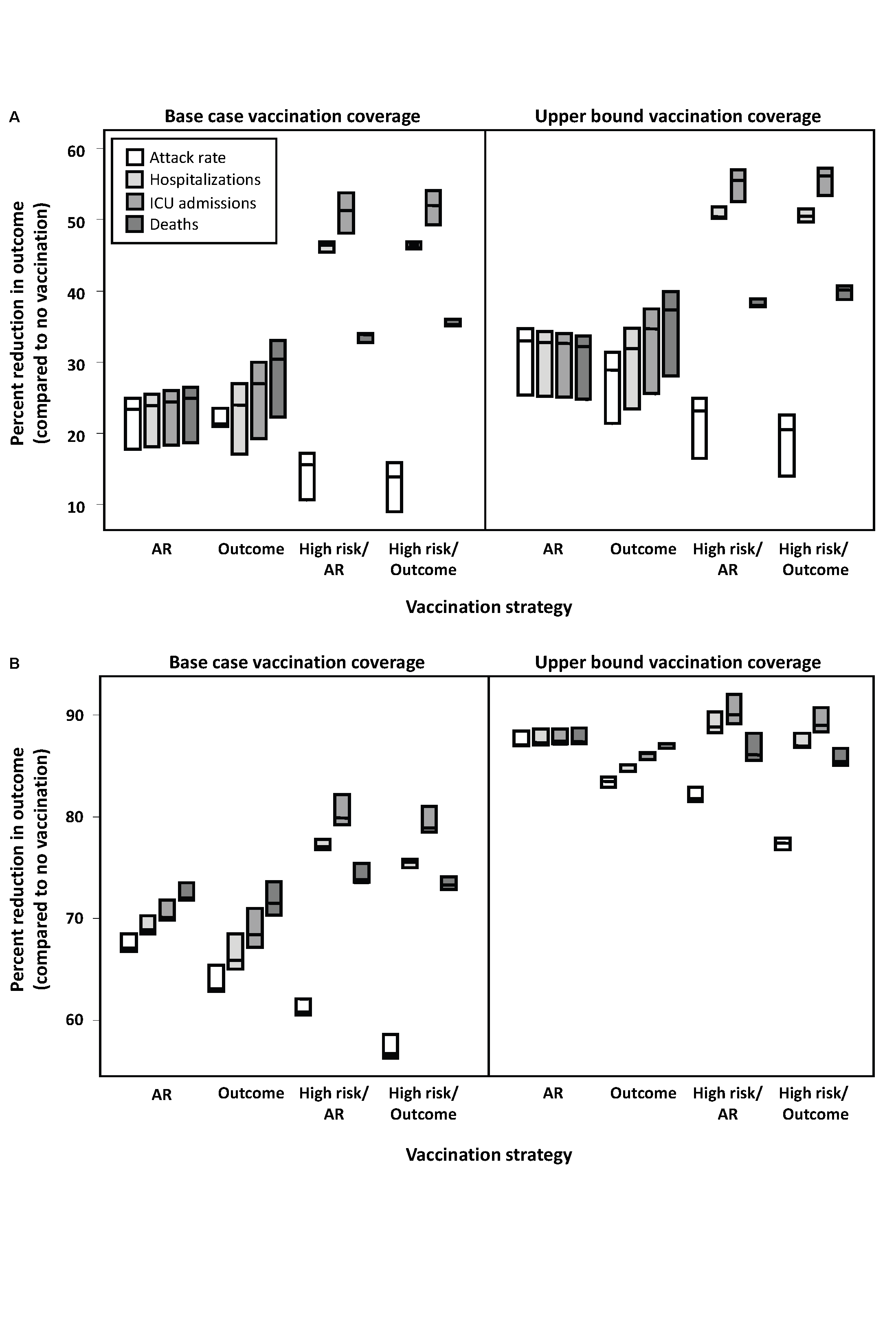

Supplement: Figure S2 — Impact of a single dose vaccination schedule on percent reduction in attack rate, hospitalizations, ICU admissions, and total deaths, relative to no vaccination. The effectiveness of different strategies was evaluated assuming an epidemic peak in (A) November, 2009 or (B) January, 2010. Results for October, 2009 and December, 2009 were similar to November, 2009 and January, 2010, respectively, and are not shown. Vaccination campaigns began on November 15, 2009, with vaccine conferring a protective effect immediately after administration of a single dose. The impact of vaccination coverage is also shown, with base case rates representing the lower bound of vaccine uptake in the Canadian population, compared to likely upper limits of vaccine uptake. The midpoint of the boxes represents the median percent reduction in the outcome of interest, with the upper and lower bounds representing the maximum and minimum reductions, respectively, under varying assumptions of pre-existing immunity in individuals aged ≥53 (i.e., 30%, 50%, or 70%). Details of the different vaccination strategies (AR, Outcome, High risk/AR, High risk/Outcome) are outlined in the Methods. (0.67 MB TIF) [file pone.0010520.s003.tif]

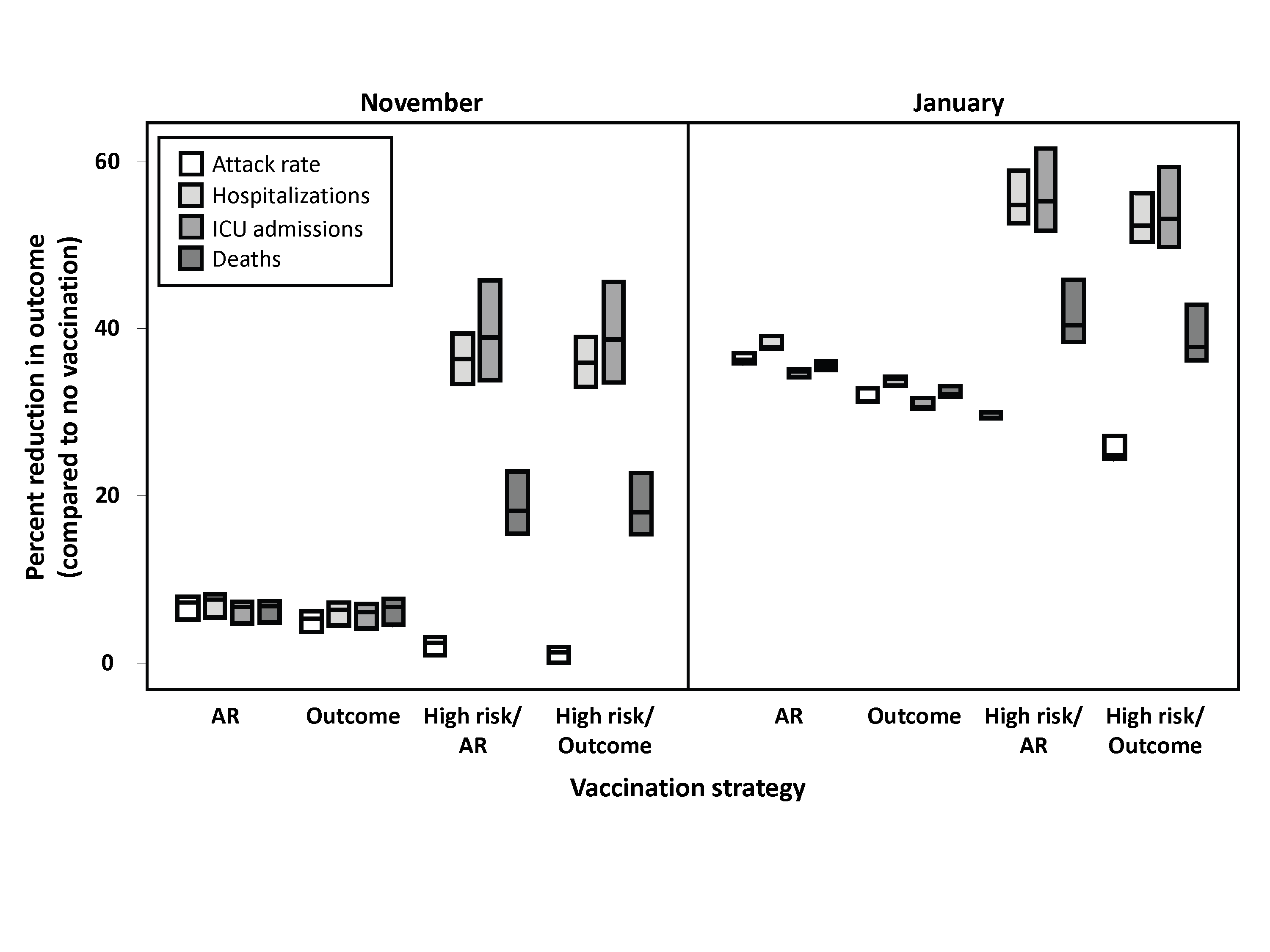

Supplement: Figure S3 — Impact of limited vaccine supply on percent reduction in attack rate, hospitalizations, ICU admissions, and death, relative to no vaccination. The effectiveness of different strategies was evaluated assuming an epidemic peak in November, 2009 or January, 2010, with vaccination campaigns initiated on November 15, 2009. Results for October, 2009 and December, 2009 were similar to November, 2009 and January, 2010, respectively, and are not shown. Enough vaccine was available to vaccinate six million individuals, with an equal number of doses available each week of the campaign. The midpoint of the boxes represents the median percent reduction in the outcome of interest, with the upper and lower bounds representing the maximum and minimum reductions, respectively, under varying assumptions of pre-existing immunity in individuals aged ≥53 (i.e., 30%, 50%, or 70%). Details of the different vaccination strategies (AR, Outcome, High risk/AR, High risk/Outcome) are outlined in the Methods. (0.52 MB TIF) [file pone.0010520.s004.tif]

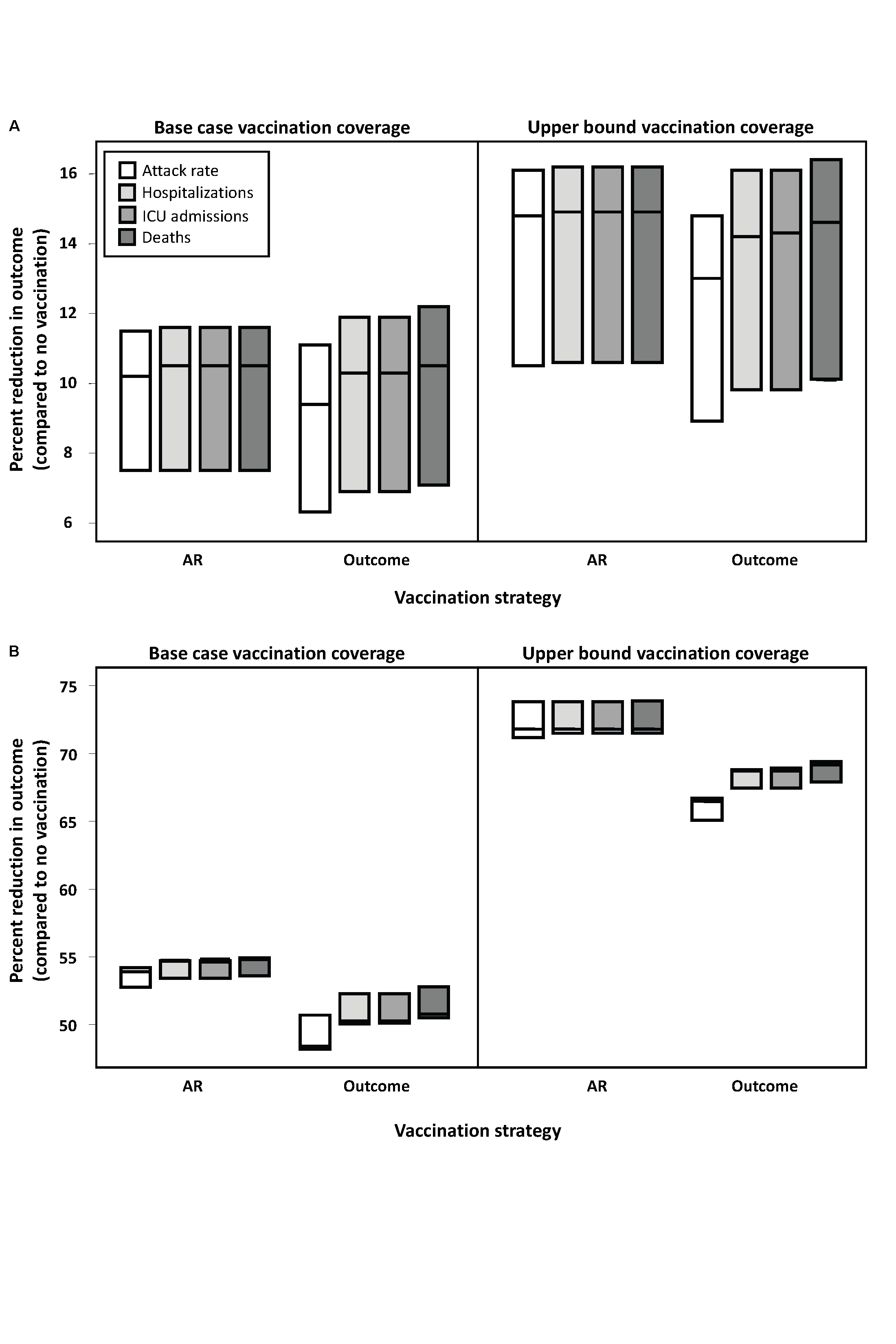

Supplement: Figure S4 — Comparison of effectiveness of attack rate- and outcome-based strategies using alternate estimates of hospitalization, ICU admission, and mortality. Percent reduction in outcomes relative to no vaccination was evaluated assuming an epidemic peak in (A) November, 2009 or (B) January, 2010, with vaccination campaigns initiated on November 15, 2009. Resultsfor October, 2009 and December, 2009 were similar to November, 2009 and January, 2010, respectively, and are not shown. The impact of vaccination coverage is also shown, with base case rates representing the lower bound of vaccine uptake in the Canadian population, compared to likely upper limits of vaccine uptake. The midpoint of the boxes represents the median percent reduction in the outcome of interest, with the upper and lower bounds representing the maximum and minimum reductions, respectively, under varying assumptions of pre-existing immunity in individuals aged ≥53 (i.e., 30%, 50%, or 70%). Details of the vaccination strategies are outlined in the Methods. (0.76 MB TIF) [file pone.0010520.s005.tif]
